# Development and characterization of fenticonazole nitrate-loaded cubogel for the management of vaginal candidiasis

**DOI:** 10.1016/j.ijpx.2025.100355

**Published:** 2025-07-10

**Authors:** Sadek Ahmed, Osama Saher, Heba Attia, Abdurrahman M. Fahmy, Islam M. Adel

**Affiliations:** a Department of Pharmaceutics and Industrial Pharmacy, Faculty of Pharmacy, Cairo University, Kasr El-Aini Street, Cairo 11562, Egypt; b Department of Laboratory Medicine, Karolinska Institute, Stockholm, Sweden; Department of Cellular Therapy and Allogeneic Stem Cell Transplantation (CAST), Karolinska University Hospital Huddinge and Karolinska Comprehensive Cancer Center, Stockholm, Sweden; c Department of Microbiology and Immunology, Faculty of Pharmacy, Cairo University, Kasr El-Aini Street, Cairo 11562, Egypt

**Keywords:** Cubosomes, Fenticonazole nitrate, Cubogel, Confocal laser microscopy, Ex vivo permeation, Histopathology

## Abstract

Vaginal candidiasis is a medical condition that affects large majority of the women at least once in their lifetime. The condition manifests with itching, irritation, and discharges which is troublesome for women during mundane activities. The purpose of this research was to formulate and evaluate the physicochemical properties and drug permeation of Fenticonazole-loaded cubogel through vaginal mucosa. Concisely, the drug-loaded cubosomes were prepared via hot dispersion emulsification technique. Following, the percent drug entrapment efficiency, particle size, polydispersity index, and zeta potential of the cubosomes were determined. Optimization criteria involved maximizing entrapment efficiency (EE %) and zeta potential (ZP), while maintaining a nanoscale particle size to ensure colloidal stability. The optimized formulation exhibited a high desirability score of 0.933 with EE % of 85.32±2.34 %, PS of 169±0.85 nm, PDI of 0.29±0.02, and ZP of −24.40±1.27 mV. In addition, 86.77±3.79 % of Fenticonazole nitrate was released from the optimum cubosomal formulation after 8 h. Cubical nanovesicles were revealed via transmission electron microscope while infrared spectroscopy revealed the lack of interaction between the used components. Stability was unchanged upon storage for three months. The rheogram of the optimum formulation-loaded cubogel suggested a shear-thinning behavior. Additionally, the optimum cubogel demonstrated higher biofilm inhibitory effect compared to the drug suspension. Similarly, both, ex vivo permeation and confocal laser scanning, suggested the enhanced vaginal epithelium permeability and the deeper vaginal mucosa penetration of the optimum cubogel, compared to the drug suspension and aqueous Rhodamine B carbopol gel, respectively. Histopathological assessment concluded with the safety of the cubogel on the vaginal mucosal epithelium and underlying tissue.

## Highlights

• A novel Fenticonazole nitrate cubogel was developed to enhance vaginal drug delivery.
• Optimized cubosomes showed high entrapment (85.3%), small size (169 nm), low PDI (0.29), and stable zeta potential (−24.4 mV).
• The system exhibited biphasic release, uniform morphology (TEM), and no drug-excipient interaction (FTIR).
• Cubogel enhanced antimicrobial action, biofilm inhibition, and mucosal penetration (CLSM).
• Histological analysis confirmed the cubogel’s safety on vaginal tissue.

## Graphical Abstract

**Figure:**
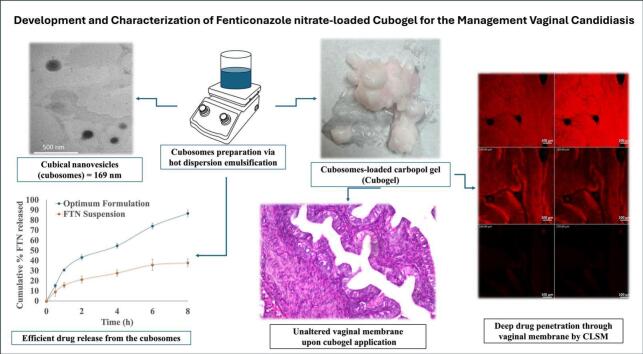

## 1 Introduction

Vaginal candidiasis is a troublesome medical condition characterized by inflammation of the vagina, commonly affecting women of childbearing age. Not only that the underlying condition affects nearly 70 % of the childbearing women population at least once in a lifetime, but also vaginal candidiasis has relatively high recurrence rates (40–50 % of initially affected women) (de Cássia Orlandi Sardi et al., 2021; Hussen et al., 2024). Although the infection is usually superficial, if not properly and promptly treated, it becomes invasive with symptoms ranging from simple itching and redness around the vagina area to thick discharges and painful urination (Darwesh et al., 2024; Elkanayati et al., 2022). *Candida albicans* is responsible for the majority of cases (90 %), while minor cases are caused by *Candida glabrata*, and *Candida parapsilosis* (Sawant et al., 2025). Common treatment involves the utilization of antifungal agents in the form of tablets, creams, gels, and suppositories. However, the poor bioavailability of many orally administered agents, the abundance of systemic side effects for systemically intended dosage forms, poor patient compliance, and vaginal discharges of topical dosage forms limit the usefulness of traditional medications (Ahmed et al., 2025c; Chayachinda et al., 2024; Kumar et al., 2022). Systemic side effects range from simple symptoms as nausea, vomiting, and abdominal discomfort to severe conditions represented with hepatitis, lupus-like syndrome, and Stevens Johnson syndrome (Lugo and Steinbach, 2023; Werth, 2012). Moreover, repeated oral application resulted in the development of drug resistance and, hence, lack of benefit (Bano et al., 2024; Helal et al., 2025).

Fenticonazole nitrate (FTN) is a synthetic imidazole derivative with broad-spectrum antifungal agent used for wide range of fungal infections throughout the body (Ji et al., 2025). FTN exhibits a concentration-dependent activity against different fungi species (Mohammed et al., 2024). At low concentration, it inhibits ergosterol production by targeting lanosterol 14α-demethylase and exerts a fungistatic action (Albash et al., 2022a). On the other hand, at higher concentrations, FTN was found to directly destabilize and damage fungal cell membranes and increase membrane permeability, effectively leading to fungicidal action (Campos et al., 2018). Additionally, FTN exerts broad antibacterial properties against gram positive bacteria linked with secondary vaginal infections (Albash et al., 2022b). Furthermore, owing to its high lipophilicity, FTN enhances tissue penetration and retention, allowing it to maintain therapeutic concentrations in mucosal environments (Albash et al., 2021; Mohammed et al., 2024). Such properties make it suitable for local delivery systems, including vaginal application. Despite its proven efficacy and safe profile, and in similarity with many imidazole anti-fungal agents, FTN suffers from poor bioavailability owing to low aqueous solubility (< 0.1 mg/mL)(Albash et al., 2022b; Albash et al., 2020).

Nanotechnology emerged as a promising solution to counteract the major setbacks of traditional dosage forms. Cubosomes are nanoparticles, exhibiting characteristic cubic structures, and formed by the emulsification of amphiphilic lipids in water in the presence of a stabilizer, to impart colloidal stability (Eldeeb et al., 2019). Overall, cubosomes exhibit small particle sizes (in the nano range), large surface areas, adequate biodegradability, noticeable mucoadhesive properties, all of which suggest their potential for controlled vaginal drug delivery (Said et al., 2021).

FTN was loaded into cubosomes to assess the benefits of a novel approach to tackle the abovementioned limitations of FTN and traditional dosage forms. Owing to its biodegradability, bioadhesiveness, biocompatibility, minimal toxicity, and its ability to self-assemble into bicontinuous liquid crystalline structures, Glyceryl monooleate (GMO) is the most widely studied amphiphilic lipid in the preparation of cubosomes (Abdelaziz et al., 2023; Milak and Zimmer, 2015). To stabilize the initial primary emulsion, a mixture of Brij 92 and Tween 80 (T80) was used to achieve optimal HLB balance. Fenchone was added to synergize the benefits of FTN in vaginal candidiasis as it was proven to exert antifungal, anti-inflammatory, and wound-healing activities (Bashir et al., 2023). More specifically, in a study performed by Ahmad et al., fenchone exhibited fungicidal activities against miconazole-resistant *Candida albicans* via inhibiting the biofilm formation and damaging fungal cell wall in a dose-dependent manner (Ahmad et al., 2022).

In this research, FTN-loaded cubosomes were prepared via hot dispersion emulsification technique. The properties of the prepared FTN-loaded cubosomes were then evaluated to ascertain their potential to treat vaginal candidiasis. The selected variables; entrapment efficiency (%EE), particle size (PS), polydispersity index (PDI), and zeta potential (ZP)—were evaluated to identify the optimum cubosomal formulation, which demonstrated %EE of 85.32±2.34 %, PS of 169±0.85 nm, PDI of 0.29±0.02, and ZP of −24.4±1.27 mV. Additional characterization revealed a biphasic FTN release pattern (86.77±3.79 % over 8 h), cubical nanoparticle morphology via TEM, and no component interaction based on FTIR. Stability was confirmed over 3 months with no significant parameter changes. Incorporation into a carbopol gel yielded a pseudoplastic, shear-thinning system suitable for patient application. Compared to FTN suspension, the cubogel exhibited more potent antibacterial and antifungal activities as well as superior biofilm inhibition. In vivo studies confirmed enhanced vaginal mucosal permeability via confocal laser scanning microscopy and affirmed the safety of the formulation through histopathological evaluation relative to untreated controls.

## 2 Materials and methodology

### 2.1 Materials

FTN (≥99 % purity, molecular weight 518.41 g/mol) was provided by Andalous Pharmaceutical Co. GMO (90 % purity, molecular weight 356.54 g/mol), Brij 92 (≥99 % purity, molecular weight 518.41 g/mol), (−)-fenchone (≥98 % purity, molecular weight 152.23 g/mol) were all procured from Sigma Aldrich, St. Louis, USA. The semipermeable dialysis cellulose membrane (molecular weight cutoff 14,000 g/mol) was acquired from Sigma Aldrich, St. Louis, USA. Other chemicals and solvents used were of analytical grade.

### 2.2 Experimental design

A 2^3^ full factorial study design was selected to evaluate the influence of varying specific formulation parameters on the properties of the prepared cubosomes using Design Expert® software (version 13, Stat-Ease, Inc., Minneapolis, MN, USA). The investigated variables were GMO: Brij 92 (X_1_), percentage of lipid phase as to the entire formulation weight (X_2_), and T80: drug (X_3_). The responses were chosen to be %EE (Y_1_), PS (Y_2_), PDI (Y_3_), and ZP (Y_4_). Table 1. highlights the chosen statistical design along with the selected variables, their levels, and the corresponding responses.

**Table 1:** 2^3^ full factorial experimental design highlighting different levels of independent variables and targeted optimization criteria for the responses.

Independent variables	Levels	
X_1_: GMO: Brij 92	2	4
X_2_: % lipid content	5 %	10 %
X_3_: T80: FTN	10	20
Responses	Optimization target	
Y_1_: %EE	Maximize	
Y_2_: PS	Maintain in range	
Y_3_: PDI	Maintain in range	
Y_4_: ZP	Maximize (absolute values)	


Abbreviations: GMO, Glycerol monooleate; T80, Tween 80; FTN, Fenticonazole; %EE, % entrapment efficiency; PS, Particle size; PDI, Poly dispersity index; ZP, Zeta potential.

### 2.3 Preparation of FTN-loaded cubosomes

FTN-loaded cubosomes were prepared via hot dispersion emulsification method as follows (Eldeeb et al., 2019). Initially, an organic lipid phase of FTN, GMO, Brij 92, and Fenchone was prepared via proper mixing and melting on a hot plate stirrer (Magnetic stirrer, Wise Stir, Daihan Scientific Co., Ltd., Korea) at 1500 rpm and 70${}^{{}^{\circ}}C$. At the same time an aqueous phase of T80 in distilled water was prepared and heated to the same temperature. Once completely melted, the aqueous phase was added dropwise to the organic one under continuous stirring. Finally, the resulted dispersion was left under stirring condition until temperature reached ambient temperature (for 2 h) before the mixture was properly stored for further inspection. Table 2. Showcases different compositions of the prepared cubosomes.

**Table 2:** Composition and characterization of the prepared FTN-loaded cubosomes.

Analsyis	Composition			Characterization					
	X_1_: GMO: Brij 92 (*w*/w)	X_2_: lipid content (%w/w)	X_3_: T80: FTN (w/w)	Y_1_: %EE	%DC	%DL	Y_2_: PS	Y_3_: PDI	Y_4_: ZP
1	2	5	20	68.51 ± 2.37	90.71 ± 1.09	0.96 ± 0.000	154.70 ± 3.68	0.12 ± 0.00	−21.20 ± 4.24
2	4	10	10	83.11 ± 1.96	91.55 ± 2.87	0.74 ± 0.000	149.60 ± 0.14	0.18 ± 0.01	−20.90 ± 4.10
3	2	10	10	79.68 ± 2.49	93.09 ± 0.49	0.71 ± 0.000	144.55 ± 5.59	0.17 ± 0.01	−20.45 ± 3.04
4	2	5	10	70.48 ± 3.49	93.79 ± 0.89	1.14 ± 0.001	140.15 ± 0.92	0.15 ± 0.02	−19.00 ± 0.85
5	4	10	20	85.32 ± 2.34	94.91 ± 1.09	0.70 ± 0.000	169.00 ± 0.85	0.29 ± 0.02	−24.40 ± 1.27
6	4	5	10	75.64 ± 2.67	92.60 ± 0.20	1.22 ± 0.000	142.60 ± 0.28	0.12 ± 0.01	−20.35 ± 0.07
7	4	5	20	77.51 ± 3.13	93.02 ± 3.17	1.08 ± 0.000	156.00 ± 1.13	0.19 ± 0.02	−21.95 ± 5.30
8	2	10	20	81.73 ± 1.58	92.81 ± 1.88	0.67 ± 0.000	164.10 ± 2.55	0.12 ± 0.00	−22.15 ± 4.31

FTN and Fenchone were added to all formulations at constant amounts (5 mg each).

Data are represented as mean ± SD.

Lipid phase was composed of GMO, Brij 92 and water was added in variable amounts as to make the total formulation weight 5000 mg.

Abbreviations: FTN, Fenticonazole; GMO, Glycerol monooleate; T80, Tween 80; %EE, %entrapment efficiency; %DC, %Drug content; %DL, %Drug loading; PS, Particle size; PDI, Polydispersity index; ZP, Zeta potential.

### 2.4 Characterization of the prepared FTN-loaded cubosomes

#### 2.4.1 Determination of percentage entrapment efficiency (%EE), percentage drug content (%DC), and percentage drug loading (%DL)

For %EE, accurately measured sample volume (1 mL; equivalent to 1000 μg FTN) of the freshly prepared formulation was placed in epindorff tube and centrifuged (Model 8880, Centurion Scientific Ltd., W. Sussex, UK) for 1 h at 21000 rpm and 4${}^{{}^{\circ}}C$. The supernatant was taken, properly diluted in methanol, and assayed for FTN content spectrophotometrically (model UV-1601 PC; Shimadzu, Kyoto, Japan) at 252 nm (R^2^ = 0.9997). The following equation was used (Ahmed et al., 2024a; Ardhi and Schreiner, 2024).

$$\%\mathrm{EE}=\frac{\text{total amount of the drug}-{\text{free drug}}^{"}\text{drug in}\;{\text{the supernatant}}^{"}}{\text{total amount of the drug}}\times 100$$

For %DC and %DL, a methanolic solution of the sample volume (0.5 mL; equivalent to 500 μg FTN) was prepared and measured for FTN content spectrophotometrically at 252 nm. %DC and %DL were calculated as follows (Adel et al., 2023; Ahmed et al., 2025a; Herdiana et al., 2024).

$$\%\mathrm{DC}=\frac{\text{actual drug amount}}{\text{added drug amount}}\times 100$$

$$\%\mathrm{DL}=\frac{\text{actual drug amount}}{\text{actual drug amount}+\text{weight of used polymers}}\times 100$$

#### 2.4.2 Measurement of particle size (PS), polydispersity index (PDI), and zeta potential (ZP)

A drop of a freshly prepared formulation (50 μL) was properly diluted with distilled water (1: 100), thoroughly vortexed, then measurements were carried at 25 °C and at a backscattering angle of 173° using Malvern ZetaSizer Nano ZS (Malvern Instruments, Worcestershire, UK).

### 2.5 Selection of the optimum cubosomal formulation

The optimization was carried out using Design-Expert® software (Stat-Ease Inc., USA), with the goal of maximizing entrapment efficiency (EE%) and zeta potential (ZP) while achieving a nanoscale particle size to ensure colloidal stability. ANOVA was conducted to evaluate the statistical significance of formulation variables and their interactions. A desirability function approach was then applied, and the formulation with the highest overall desirability score—0.933—was selected as the optimum. This formulation was subsequently prepared and characterized to validate the model's predictions.

### 2.6 Characterization of the optimum cubosomal formulation

#### 2.6.1 In vitro release study

Drug release behavior was studied via dialysis bag method (Ahmed et al., 2025; Das et al., 2011). Briefly, an accurate volume of the optimum formulation (1 mL; equivalent to 1000 μg FTN) was placed in a one-sided, tightly closed, semipermeable dialysis bag that was presoaked in distilled water overnight. Following, the bag was carefully closed from the loose end and immediately placed in a 90 mL glass bottle filled with 50 mL of phosphate buffer saline (PBS; pH 4.5)- absolute ethanol mixture (3: 1 *v*/v). The bottles were placed in a water bath shaker (Unimax, IKA, Germany) adjusted thermostatically at 37±0.2${}^{{}^{\circ}}C$ and allowed to run at 100 rpm. At select time intervals (0.5, 1, 2, 4, 6, and 8 h), 3 mL samples were withdrawn and assayed spectrophotometrically for FTN content at 252 nm. The withdrawn samples were replaced with equal fresh volumes of the release medium. The resulting data was plotted as % cumulative drug release vs time. Data obtained was analyzed to define the model of drug release (zero order, first order, and Higuchi diffusion models).

#### 2.6.2 Transmission electron microscopy (TEM)

The morphological characteristics of the optimum formulation were studied using TEM (JEOL RI 2100, Frankfurt, Germany). A drop of the freshly prepared selected formulation was properly diluted with distilled water and vortexed for 5 min. Following, a drop of the dispersion was fixed on a copper grid and stained with 0.1 % phosphotungstic acid. The assembly left to air-dry for 15 min before being scanned at 70 kV (Fahmy et al., 2025; Younes et al., 2024).

#### 2.6.3 Fourier-transform infrared spectroscopy (FT-IR)

FT-IR spectra for the optimum cubosomal formulation as well as FTN were recorded in order to identify any possible interactions on a molecular level (Ahmed et al., 2024b). The optimum formulation was frozen (−20${}^{{}^{\circ}}C$), then lyophilized (−45${}^{{}^{\circ}}C$, Novalyphe-NL 500 freeze-dryer, Savant Instruments, NY, USA). Following, an accurate amount (2 mg) of the lyophilized formulation was pressed into a disc after proper mixing with dry potassium bromide. The sample was scanned at a range of 4000–400 cm^−1^ using FT-IR spectrophotometer (Model, 8400 s Shimadzu, Kyoto, Japan). Same procedure was carrier for FTN.

#### 2.6.4 Short term stability study

The optimum cubosomal formulation was characterized in terms of %EE, PS, ZP, as well as in vitro release behavior prior to 3-month storage in a tightly sealed vial at 4–8${}^{{}^{\circ}}C$. Post 3-month storage, the formulation was reassessed for the same parameters and results of %EE, PS, and ZP were compared with One-Way ANOVA. Meanwhile, release behavior was compared via calculation of similarity factor (*f*_*2*_) according to the following equation (Adel et al., 2021).

ƒ_2_ = 50.log [{1 + ($\frac{1}{n}$) ∑${}_{t=1}^{n}$ (R_t_-T_t_)^2^}^-0.5^.100].

Where n is the sampling number while R_t_ and T_t_ refer to % drug released after certain time t from fresh and stored samples, respectively.

### 2.7 Preparation of FTN-loaded cubogel

The optimum formulation was loaded into carbopol-based gel for further testing. Immediately on preparation, the dispersion of the optimum formulation was warmed to 40${}^{{}^{\circ}}C$, carbopol was then slowly sprinkled (to a final concentration of 1 % *w*/*v*) under magnetic stirring (Magnetic stirrer, Wise Stir, Daihan Scientific Co., Ltd., Korea) at 300 rpm until completely dissolved and left to cool and form clear gel at room temperature overnight. The prepared gel was further tested.

### 2.8 Rheological study

The rheological behavior of the prepared cubogel was evaluated using the cone and plate rheometer (Brookfield DV3THB cone/plate rheometer, spindle CPE-40, and RheocalcT software, v1.1.13, Middleboro, MA, USA) where a temperature of 25±2${}^{{}^{\circ}}C$ was kept constant via surrounding the outer cylinder with a water bath (PolyScience model 9006, Niles, IL, USA). Briefly, an accurately weighed sample (0.5 g) was placed on the plate, allowed to rotate at an increased speed over a range of 0.5–100 rpm with a 10-s speed-shift interval. The obtained data was used to plot the rheogram and the Ostwald de Waele model was applied to study the flow behavior of the FTN-loaded cubogel (Nemr et al., 2021).

$$\tau =K\gamma^{n}$$

where τ represents the shear stress while γ represents the shear strain. K is the power law or consistency constant, and n is the power law index. According to n value, the flow behavior can be determined. Generally, *n* = 1 refers to Newtonian fluid, *n* < 1 usually indicates shear-thinning behavior or pseudoplastic fluid, and *n* > 1 indicates shear-thickening behavior or dilatant fluid (Guo, 2021). To identify the type of non-Newtonian flow, R^2^ was calculated for Bingham's, Casson's, and Carreau's models (Fahmy et al., 2018; Hegazy et al., 2025).

### 2.9 Microbiological assay

*Candida albicans* studies are frequently conducted at 28±2${}^{{}^{\circ}}C$ because this temperature range aligns with established mycological guidelines for fungal cultivation, balances optimal yeast-phase growth with minimized bacterial overgrowth, and preserves reproducible phenotypes (e.g., planktonic *vs* filamentous forms). Specifically, CLSl standards recommend this range for fungal media to ensure consistent recovery and growth on selective agars (e.g., Malt Extract Agar) and Sabouraud-Dextrose Agar, and avoid excessive hyphal induction that occurs at higher temperatures (CLSI, 2004).

#### 2.9.1 Determination of minimum inhibitory concentration (MIC)

The broth microdilution method was adopted to determine the minimum inhibitory concentration (MIC), following the guidelines of the Clinical and Laboratory Standards Institute (CLSI, 2018). Both the optimum formulation as well as the drug suspension were prepared in a concentration range of 500–0.488 μg/mL in a total volume of 150 μL, by a two-fold dilution technique, in double strength Sabouraud Dextrose Broth (SDB), and dispensed into sterile 96-well microtiter plates with a characteristic U-shaped bottom. Thirty microliters of *Candida albicans* standard strain (ATCC 60193) suspension (inoculum size of 10^5^–10^6^ CFU/mL) was then added to each well. A control test for the yeast growth and another control test for the culture media sterility were adopted. Biological and technical triplicates of the experiment were conducted. After incubating the microtiter plates at 28±2${}^{{}^{\circ}}C$ for 48 h, the plates were examined visually and by measuring each well's turbidity absorbance at a wavelength of 600 nm in a 96-well plate reader (Biotek, Synergy 2 SLFA model, USA). The MIC was determined as the lowest concentration showing no observable growth.

#### 2.9.2 Determination of minimum fungicidal concentration (MFC)

To determine the minimum fungicidal concentration (MFC) of both the drug suspension and the optimum formulation, two-fold serially diluted preparations were incubated with 30 μL yeast inoculum suspension (inoculum size of 10^5^–10^6^ CFU/mL) in 96-well plates at 28±2${}^{{}^{\circ}}C$ for 48 h. Following incubation, 10 μL aliquots of the yeast-preparation mixture were transferred from each well, till the well corresponding to MIC, and were spotted on the surface of Sabouraud Dextrose Agar (SDA). The plates were then incubated at 28±2${}^{{}^{\circ}}C$ for another 48 h. The MFC was expressed as the lowest concentration that showed no visible fungal growth. The test was performed in biological and technical triplicates.

#### 2.9.3 Determination of biofilm inhibitory effect

The inhibitory effects of the drug suspension and the optimum formulation on the tested *Candida albicans* strain biofilm formation were evaluated using the standardized crystal violet assay. An accurately measured 80 μL of yeast suspension (10^8^ CFU/mL in SDB) was added to 80 μL of either the drug suspension or the optimum formulation in non-pyrogenic, sterile 96-well polystyrene plates with flat bottoms. Final well concentrations corresponded to serial dilutions of ^1^/_16_, ^1^/_8_, ^1^/_4_, and ^1^/_2_ X, where X represents the predetermined MIC. Negative controls (sterility verification controls) and positive controls (untreated yeast biofilm growth) were included (O'Toole, 2011; Van Dijck et al., 2018). Following a 48-h static incubation at 28±2${}^{{}^{\circ}}C$, the optical density (O.D.600) of the yeast culture was measured using an automated spectrophotometric plate reader (Biotek Synergy 2 SLFA model, USA). Supernatants were gently removed, and the wells were double rinsed with sterile PBS (pH 7.4), then thoroughly air-dried. The dried adhered biofilms were stained with 180 μL of 0.5 % *w*/*v* crystal violet for 30 min at room temperature. Excess stain was then removed by washing the plates three times with sterile distilled water, followed by thorough air-drying. To solubilize the stained biofilm, 200 μL of 95 % ethanol was added to each well, followed by 15 min of incubation with orbital shaking (110 rpm) at room temperature. The absorbance of the solubilized crystal violet-stained biofilm was measured at O.D.570 using the aforementioned plate reader. Readings were normalized to the O.D.600 absorbance values of planktonic cultures to account for variations in baseline growth. The experiment was repeated three independent times and done in technical replicates. The biofilm inhibition percentage was calculated according to the following equation:

$$\text{Biofilm inhibition}\%=\frac{\mathrm{OD}\;\text{Control}-\mathrm{OD}\;\text{Test}}{\mathrm{OD}\;\text{Control}}\times 100$$

The statistics were performed in version 4.1.2 of R and visualized in Rstudio (Racine, 2012).

### 2.10 Animal studies

#### 2.10.1 Ex vivo permeation study

##### 2.10.1.1 Vaginal membrane preparation

Six adults (randomly divided into two groups; *n* = 3), recently born, female albino rabbits, weighing 2±0.2 kg, were used for the experiment. Initially, the rabbits were anesthetized with i.m. injection of ketamine (35 mg/kg) and xylazine (5 mg/kg). Following, vaginal membrane samples was carefully excised from anesthetized rabbits, proteins were denaturized and fatty layers removed using isopropyl alcohol, and double-cleansed with saline and deionized water. The fat-free vaginal membranes were stored under controlled conditions and a temperature of −20${}^{{}^{\circ}}C$.

##### 2.10.1.2 Experimental procedure

Permeation study was performed using a modified Franz-diffusion cell as follows. The excised vaginal membrane was fixed between the donor and the recipient compartments in a way where upper layer was directed to the donor cell. A specific amount of FTN-loaded cubogel (2 g; equivalent to 1000 μg FTN) was placed on the upper side of the membrane (donor compartment). The receptor medium, facing the other side (area of 0.785 cm^2^), consisted of 25 mL mixture of PBS 4.5: absolute ethanol (3: 1 *v*/v). The modified diffusion cell was set under magnetic stirring (100 rpm) and at controlled temperature (37±0.2${}^{{}^{\circ}}C$). At predefined time intervals, 1 mL sample was withdrawn from the receptor medium and replenished with exact same volume of fresh receiver medium. Furthermore, all samples were filtered through a 0.22 μm nylon filter prior to measurement against a blank solution containing the release medium, ensuring that the results were free from any extraneous interference. Withdrawn samples were assessed for FTN content using UV spectrophotometer at 252 nm. The same procedure was applied to plain FTN gel for comparison purposes where the upper membrane layer was in contact with 2 mL FTN suspension (equivalent to 1000 μg). The results were interpreted in the form of cumulative amount of FTN permeated per unit area vs time. Both the amount permeated per unit area (flux; $J_{\max}$) and the enhancement ratio (ER) were calculated as follows (El Zaafarany et al., 2010; Sayed et al., 2018).

$$J_{\max}=\frac{\text{amount of drug permeated}}{\text{time}\times \text{area}}$$

$$\mathrm{ER}=\frac{J_{\max}\;\text{of formulation}}{J_{\max}\;\text{of plain}\;\mathrm{gel}\;\left(\text{control}\right)}$$

#### 2.10.2 Confocal laser scanning microscopy (CLSM)

In order to evaluate the permeation uptake efficiency of the optimum formulation through the vaginal mucosa, CLSM was carried. Briefly, the optimum cubogel was prepared as before, except for the replacement of FTN by Rhodamine B (RhB; fluorescent material) at a concentration of 0.1 % *w*/w (treatment group). Meanwhile, an aqueous RhB solution was formulated into carbopol gel format to serve as control. In either case, the gel was applied into the vaginal cavity of a recently born female albino rabbit. After 6 h, the animal was anesthetized, decapitated, and the vaginal mucosa was excised and cleansed thoroughly with saline and deionized water. Samples were placed between glass slide and cover and investigated using CLSM (LSM 710, Carl Zeiss, Jena, Germany) where RhB fluorescence was analyzed at λ_max_ = 485 nm and 595 nm (Ahmed et al., 2025b). Images were resolved using LSM software version 4.2 (Carl Zeiss Microimaging, Jena, Germany).

#### 2.10.3 Histopathological assessment

Assessment of possible inflammatory reaction and tissue alteration due to the applied cubogel was carried using hematoxylin and eosin staining technique as follows (Layton and Suvarna, 2013). Two groups of female albino rabbits (6 rabbits, *n* = 3) were used, where one received the treatment (cubogel vaginal application) and the other served as a negative control (left untreated). Application was carried (1 mg of assigned medication) as twice daily dosage regimen for 1 week. After treatment period was over, autopsies were taken from the vagina, fixed in 10 % formol saline for 24 h, and then washed with distilled water, and finally dehydrated using serial dilutions of alcohol (methyl, ethyl, and absolute ethyl alcohols). Following, the collected specimen was cleared in xylene and soaked in paraffin in hot air over adjust at 56${}^{{}^{\circ}}C$ for 24 h. Paraffinized tissue blocks were cut using rotary LEITZ microtome (4 μm thickness), placed on glass slides, deparaffinized, and then stained with hematoxylin and eosin. Finally, slides were examined using light electric microscope (CX21Olympusmicroscope, Tokyo, Japan).

### 2.11 Statistical analysis

Statistical design and analysis were performed using Design Expert® software (version 13, Stat-Ease, Inc., Minneapolis, MN, USA). Analysis of the responses was conducted using One-Way ANOVA, setting significance level at *p* < 0.05. Between-group significance was detected by Least Significance Difference (LSD) test. All tests were conducted in triplicates (n = 3) and data was presented as mean±SD.

## 3 Results and discussion

### 3.1 Characterization of the prepared FTN-loaded cubosomes

#### 3.1.1 Determination of percentage entrapment efficiency (%EE), percentage drug content (%DC), and percentage drug loading (%DL)

%EE, %DC, and %DL data for the performed runs can be visualized in Table 2.

%EE (Y_1_) for the prepared formulations ranged between 68.51±2.37 to 85.32±2.34 % and the model equation was:

%EE=77.75+2.65A+4.71B+0.52C

**Fig. 1:**
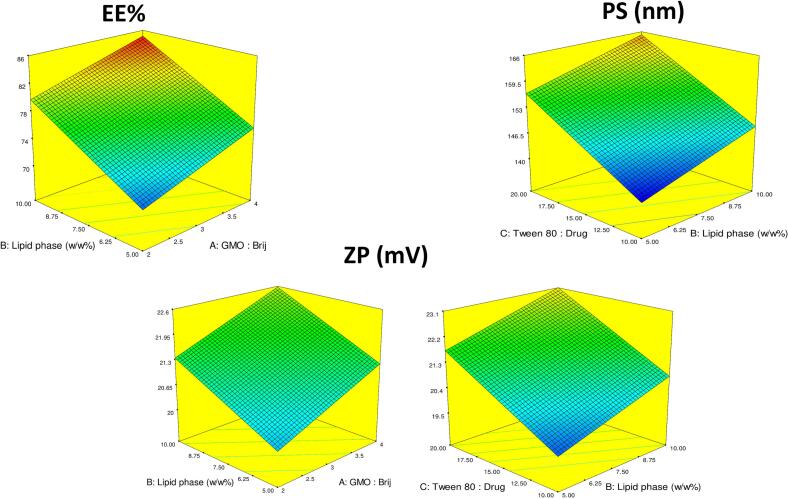
3D response plots illustrating the effect of different independent variables on the selected optimization parameters of percentage entrapment efficiency (%EE), particle size (PS), and zeta potential (ZP).

An increase in the GMO: Brij 92 ratio (Factor A) was found to significantly enhance the entrapment efficiency (%EE) (p < 0.05; Fig. 1a). This can be attributed to the unique physicochemical characteristics of glyceryl monooleate (GMO), which forms lyotropic liquid crystalline phases—particularly bicontinuous cubic phases—offering a large internal surface area and densely packed lipid structures. These features minimize drug leakage and create a favorable microenvironment for encapsulating lipophilic drugs such as FTN. Additionally, the amphiphilic nature of GMO facilitates the simultaneous incorporation of both hydrophilic and lipophilic molecules into distinct domains within the nanostructure.

Similarly, increasing the total lipid phase concentration (Factor B) also led to a significant rise in %EE (p < 0.05). A higher lipid content contributes to the formation of a greater number of vesicular units and thicker bilayer membranes, both of which enhance the capacity of the system to encapsulate and retain the drug. Furthermore, a higher lipid fraction provides an extended hydrophobic region for FTN solubilization, thus promoting greater drug loading within the cubosomal matrix (Adel et al., 2021). T80: drug had no significant impact (*p* > 0.05) on %EE.

On the other hand, the drug content (%DC) values ranged from 90.71 ± 1.09 % to 94.91 ± 1.09 %, while drug loading (%DL) ranged from 0.67 ± 0.000 % to 1.22 ± 0.000 %. These consistently high %DC values reflect minimal drug loss during the preparation process, highlighting the reproducibility and efficiency of the hot dispersion emulsification technique employed. The negligible variation also underscores the formulation's homogeneity and process robustness.

#### 3.1.2 Measurement of particle size (PS)

Overall, the PS ranged between 140.2±0.92 nm to 169±0.85 nm and the representative equation was:

PS=152.588+1.7125A+4.225B+8.3625C

The particle size (PS, Y₂) data (Fig. 1b) revealed that altering the GMO:Brij 92 ratio (X₁) did not result in a statistically significant change in PS (p > 0.05). This finding contrasts with reports by Ahirrao et al., where the PS of resveratrol-loaded cubosomes increased with higher GMO concentrations and decreased with increased surfactant content (Ahirrao and Shrotriya, 2017). The observed increase in PS was attributed to elevated viscosity at higher GMO levels, which impedes the formation of tightly packed nanostructures and promotes the aggregation of larger vesicles (Ahmed et al., 2023). Conversely, Miyamoto et al. demonstrated that incorporating Brij surfactants into nanoemulsions led to a reduction in particle size, regardless of the Brij type (Miyamoto et al., 1999). Their findings suggest that the presence of Brij molecules reduces interfacial tension and stabilizes smaller droplets during emulsification. Taken together, the opposing effects of increasing GMO (which tends to enlarge PS) and Brij 92 (which tends to reduce PS) may have counterbalanced each other in our experimental system, ultimately resulting in non-significant net changes in PS. This equilibrium further underscores the importance of carefully optimizing lipid-to-surfactant ratios in nanocarrier design.

In contrast, increasing the total lipid phase concentration (X₂) led to a statistically significant increase in PS (*p* < 0.05). This can be explained by the elevated fatty matrix density, which promotes vesicle swelling and enhances particle growth during self-assembly. Similar trends were previously reported by Adel et al., where higher lipid content yielded larger vesicles due to increased lamellarity and internal volume (Adel et al., 2021). Moreover, increasing the T80: drug ratio (X₃) also resulted in a significant (p < 0.05) enlargement in PS. This may be attributed to the incorporation of excess Tween 80 molecules into the bilayer structure of the vesicles. The intercalation of these surfactants can disrupt the compactness of the lipid bilayers, thereby increasing membrane fluidity and curvature, which leads to the formation of more expanded vesicles. Collectively, these findings highlight the complex interplay between lipid and surfactant concentrations in governing vesicle size, which in turn impacts drug release, mucosal penetration, and overall formulation performance. Understanding and controlling these parameters is essential for tailoring nanocarrier characteristics to specific therapeutic applications.

#### 3.1.3 Measurement of polydispersity index (PDI)

PDI values ranged from 0.12 to 0.29±0.02, indicating the narrow range of particle distribution (Raval et al., 2019). The coded equation for PDI was:

PDI=0.1666+0.0277A+0.0207B+0.0119C

The polydispersity index (PDI) serves as an essential indicator of particle size distribution consistency within nanocarrier systems. A lower PDI—typically approaching zero—reflects a narrow size distribution, which is desirable for ensuring formulation uniformity, physical stability, and reproducibility during scale-up and storage. As presented in Table 2, all tested formulations exhibited PDI values below 0.3, indicating a fairly consistent and monodisperse vesicular population. Analysis of variance (ANOVA) revealed that none of the independent variables significantly influenced PDI values (*p* > 0.05). Such homogeneity is particularly advantageous for mucosal drug delivery systems like vaginal cubogels, as it promotes predictable permeation behavior, reduces aggregation risk, and contributes to improved patient safety and therapeutic efficacy.

#### 3.1.4 Measurement of zeta potential (ZP)

The representative equation for ZP is as follows:

ZP=21.3+0.6A+0.675B+1.12C

ZP results (Y_4_; Fig. 1c) revealed that all of the formulations had negative surface charges (values ranged from −19±0.85 to −24.4±1.27 mV). That could be due to the ionization of the carboxylate groups on GMO and surfactant surfaces (Gagliardi et al., 2020). Increasing the levels of the studied factors led to an increase in ZP values. This can be seen on increasing GMO: Brij 92 (X_1_) in runs 1 and 7 where ZP value increased from −21.20±4.24 mV to −21.95±5.30 mV, respectively. Similar observations were noticed for lipid content (X_2_) (ZP value increased from −21.20±4.24 in run 1 to −22.15±4.31 in run 8, and also for T80: FTN ratio (X_3_) (ZP value increased from −19.00±0.85 mV in run 4 to −21.20±4.24 mV in run 1).

A zeta potential magnitude greater than ±20 mV is generally considered sufficient to maintain colloidal stability due to electrostatic repulsion forces. Thus, the observed values confirm that all formulations fall within a desirable range, minimizing the risk of vesicle aggregation during storage and ensuring long-term dispersion uniformity. The results of PS, PDI, and ZP for all formulations can be seen in Table 2.

### 3.2 Selection of the optimum cubosomal formulation

Based on the findings from the previous measurements of %EE (Y_1_), PS (Y_2_), PDI (Y_3_), and ZP (Y_4_), the cubosomal formulation in the 5th analysis (Table 1.) was selected as the optimum formulation (desirability of 0.933). The optimum formulation was composed of a lipid phase of 400 mg GMO, 100 mg Brij 92, 5 mg Fenchone, and 5 mg FTN mixed with an aqueous phase of 100 mg T80 in 4.5 mL water. The obtained results revealed that the optimum formulation had a %EE of 85.32±2.34 %, PS of 169±0.85 nm, PDI value of 0.29±0.02, and a ZP of −24.4±1.27 mV. Therefore, it was to be further tested in vitro prior to in vivo evaluation. Variation between the predicted values to the actual experimental results were less than 5 % ensuring the validity of optimization process.

### 3.3 Characterization of the optimum cubosomal formulation

#### 3.3.1 In vitro release study

**Fig. 2:**
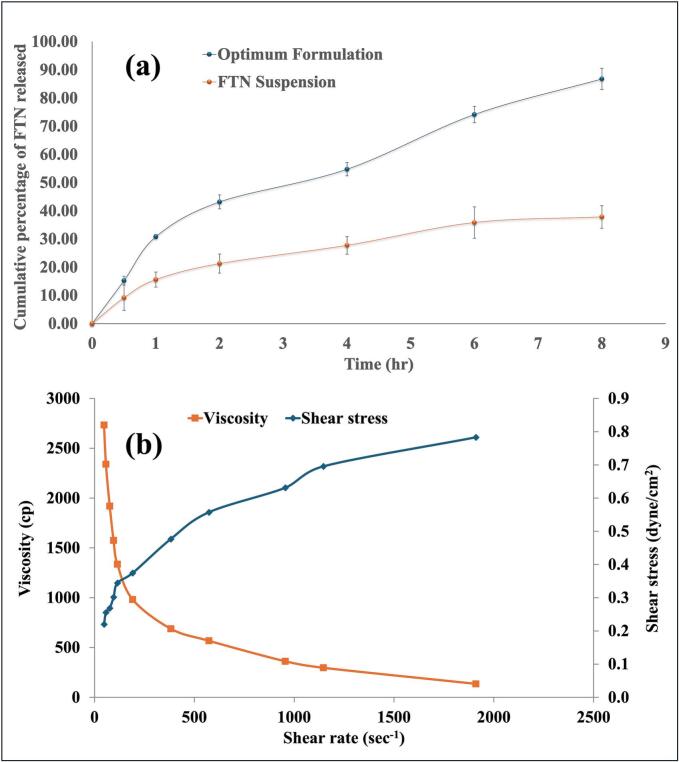
a. Release profile of fenticonazole from the optimum cubosomal formulation and its aqueous suspension, represented as % cumulative drug release against time. b. The rheogram of the formulated fenticonazole-loaded cubogel showing the shear-thinning behavior of the gel.

Fig. 2a. highlights the release profile of FTN from the optimum cubosomal formulation where a characteristic biphasic release pattern can be observed. The initial burst effect during the first 2 h (43.14±2.49 %) was caused by the release of drug molecules from the surface layers of the cubosomes while the following sustained phase (86.77±3.79 % after 8 h) was attributed to the slow diffusion of the drug from the cubosomal core. Such biphasic pattern is common for most nanoparticle-based drug delivery systems and well reported in the literature for cubosomes, niosomes, liposomes, …etc. Zhang et al. prepared combinatorial drug-loaded (cisplatin and paclitaxel) cubosomes coated with poly-ε-lysine where both the uncoated and coated cubosomes exhibited burst release effect during the first 1 h (52±2 % and 22±3 %, respectively) followed by sustained release for 6 h and 24 h, respectively (Zhang et al., 2020). Similarly, Eldeeb et al. prepared brimonidine proniosomal gel which showed an initial burst during the first 2 h (up to 60 %) followed by a sustained pattern for 24 h (Emad Eldeeb et al., 2019). The resulting release profile was compared to that of the pure drug suspension (Fig. 2a). It is worth mentioning that the drug release from the drug-loaded cubosomal formulation was superior to that of the drug suspension, indicating the enhanced efficiency of drug release from the optimum cubosomal formulation as compared to that from drug suspension (Q_8h_ = 86.77±3.79 % FTN vs 37.82±3.99 %, respectively). This finding could be due to the lower solubility of FTN in suspension than when formulated as nanoparticles (Khudair et al., 2020). Fitting the data into different release models indicated that FTN release from drug-loaded cubosomal formulation followed Higuchi's diffusion model with the highest R^2^ (0.987) vs those of zero (0.963) and first (0.973) orders. This biphasic and diffusion-controlled release profile is highly favorable for vaginal drug delivery, as it ensures a rapid onset of antifungal action followed by prolonged drug retention at the site of infection, potentially reducing dosing frequency and improving patient adherence.

#### 3.3.2 Transmission electron microscopy (TEM)

**Fig. 3:**
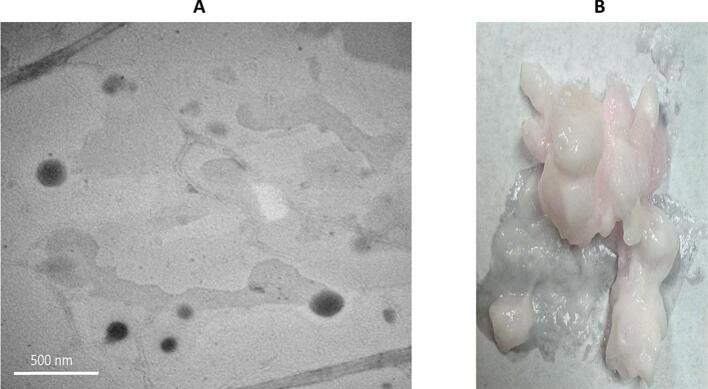
(A) TEM images of the optimum cubosomal formulation showing cubosomes, lacking any sort of aggregation. (B) Clear cubogel formulation.

Transmission electron microscopy (TEM) was utilized to gain detailed insights into the morphological and topological features of the prepared nano systems, complementing the data obtained from dynamic light scattering (DLS). As observed in Fig. 3a, TEM images revealed well-formed nanovesicles with sizes that are consistent and comparable to those measured by the zetasizer (DLS). Although the cubosomes have cubical structures, entrapment of FTN could have led to conformational changes leading to spherical outline. Similar finding was reported by Zhang et al. upon scanning both blank and drug-loaded cubosomes (Zhang et al., 2020). These findings confirm the successful formation of nanoscale vesicular systems with appropriate morphology and size, and suggest that structural adaptability upon drug loading may facilitate improved membrane interaction and diffusion, further enhancing the delivery potential.

#### 3.3.3 Fourier-transform Infrared Spectroscopy (FT-IR)

**Fig. 4:**
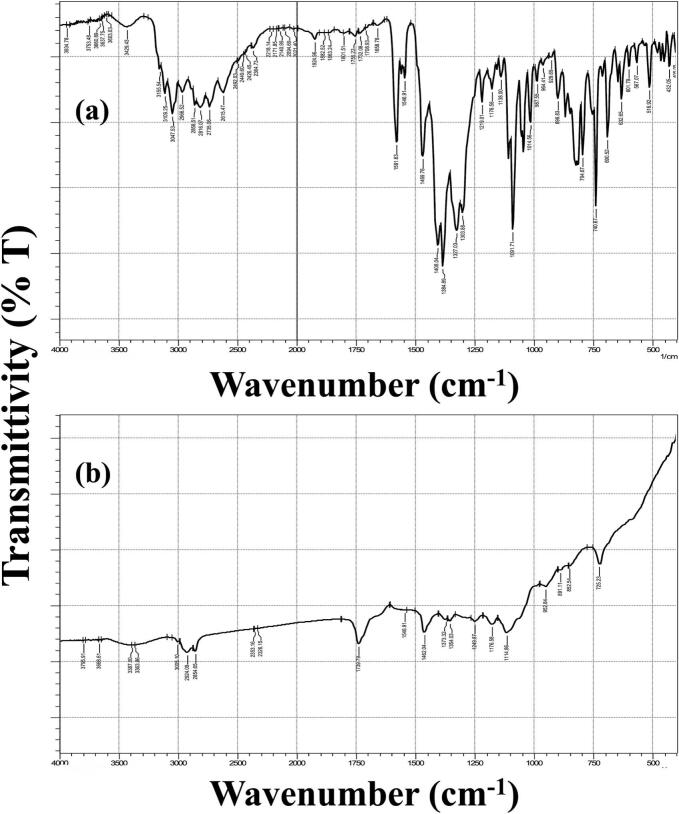
FT-IR spectra of (a) fenticonazole and (b) the optimum drug-loaded cubosomal formulation.

The FT-IR spectra can be seen in Fig. 4. In FTN spectrum (Fig. 4a.), the characteristic bands at 3047 cm^−1^ and 1581 cm^−1^ represent the stretching vibrations of N-H and C=N, respectively. Similarly, peaks can be seen at 1469 cm^−1^ (due to stretching C=C) and 1091 cm^−1^ (due to stretching aromatic ring). Overall, the FT-IR spectrum is consistent with what can be found in the literature (Ahmed et al., 2022; Castro et al., 2016). In the spectra of the optimum cubosomal formulation (Fig. 4b.), the characteristic peak of FTN due to the N-H witnessed a slight shift in wavelength to 2924 cm^−1^ which can be attributed to possible hydrogen bond formation with the abundant OH groups of GMO. Moreover, the intensity of the remainder peaks was severely reduced, suggestive of the successful entrapment of FTN by the formed cubosomes.

#### 3.3.4 Short term stability study

Following the 3-month storage period at 4–8${}^{{}^{\circ}}C$, there were no significant statistical differences in the properties of the optimum cubosomal formulation immediately after formulation and after 3 months of storage. For instance, a PS value of 167.45±6.58 nm as well as a ZP value of 28±1.48 imply no significance changes were detected (*p* > 0.05). That reassures that storage did not produce any agglomeration or surface charge alterations in the prepared nanoparticles. Furthermore, %EE was left unchanged (81.08±3.12 %; p > 0.05) confirming that storage under such conditions did not bring about any change to the stability of the cubosomal formulation. Finally, on comparison of the release profiles of the fresh and stored formulations, Q_8h_ was not significantly altered (p > 0.05), and an ƒ_2_ value of 69.05 % suggested the in vitro similarity of the two release profiles (Xie et al., 2015). Overall, the findings were strong indicatives of the minimal effects of storage over the stability of the formulated cubosomes, under the testing conditions. This confirmed stability over an extended storage period ensures that the cubosomal formulation retains its integrity, efficacy, and safety over time, which is critical for maintaining therapeutic consistency, reducing variability in patient outcomes, and ensuring user confidence in real-world clinical settings.

#### 3.3.5 Rheological study

From the rheogram in Fig. 2b. and based on the obtained n value (0.3347), one can notice the shear-thinning behavior of the cubogel indicating a pseudoplastic fluid-type gel (Ansari et al., 2020) which facilitates vaginal application. Upon applying minimal force, the cubogel would lose its structure allowing for easy application while after that and on force removal, it retains back its structure allowing for sustained drug release. Additionally, it was found that the R^2^ values for Bingham's, Casson's, and Carreau's models were 0.8936, 0.9573, and 0.9843, respectively. Concludingly, the cubogel was found to follow a non-Newtonian flow based on Carreau's model. Fig. 3b illustrates the transparent and homogeneous appearance of the cubogel formulation. These rheological properties, combined with its visual clarity, confirm the cubogel's suitability for vaginal administration, offering ease of application, strong mucosal adherence, and favorable drug release behavior.

#### 3.3.6 Microbiological assay

##### 3.3.6.1 Determination of minimum inhibitory concentration (MIC)

Broth microdilution technique was utilized to determine MIC, thereby assessing the potential improvement in antifungal action achieved through optimizing the formulation. The optimum formulation had a notably lower MIC (1.93 μg/mL) compared to that of the drug suspension (15.6 μg/mL), demonstrating a substantial enhancement in the antifungal activity of the parent drug. This significant reduction in MIC reflects not only improved drug solubility and penetration but also suggests enhanced interaction of the optimized formula with fungal cell membranes, likely due to its nanoscale size, mucoadhesive nature, and prolonged retention at the site of infection.

##### 3.3.6.2 Determination of minimum fungicidal concentration (MFC)

Similar to MIC determination, MFC was determined for drug suspension as well as the optimum formulation via broth microdilution technique. Both the drug suspension and the optimum formulation exhibited fungicidal effects after 48 h of incubation at 28±2${}^{{}^{\circ}}C$. The optimum formulation exhibited a prominently higher fungicidal activity (MFC = 3.9 μg/mL) when compared to the drug suspension (MFC = 62.5 μg/mL). This pronounced enhancement in fungicidal activity may be attributed to the enhanced membrane-disruptive effects of the optimized formula, facilitated by its nanoscale structure and prolonged mucosal residence.

##### 3.3.6.3 Determination of biofilm inhibitory effect

**Fig. 5:**
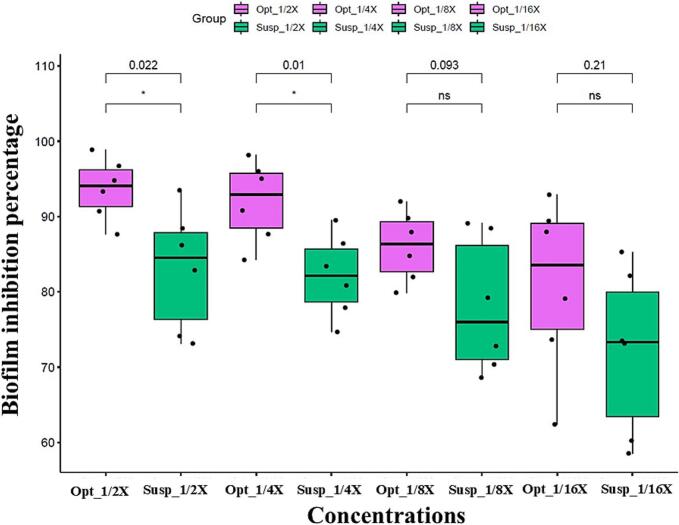
Inhibitory effect, in the form of biofilm inhibition percentage, of different concentrations (^1^/_16_, ^1^/_8_, ^1^/_4_, and ^1^/_2_ X, where X is the calculated MIC) of the optimum formulation (Opt) and drug suspension (Susp) on *Candida albicans* standard strain biofilm formation. The sub-MIC concentrations of the optimized formula were as follows: ^1^/_16_ X = 0.12 μg/mL, ^1^/_8_ X = 0.241 μg/mL, ^1^/_4_ X = 3.9 μg/mL, ^1^/_2_× = 0.965 μg/mL. The sub-MIC concentrations of the drug suspension were as follows: ^1^/_16_ X = 0.975 μg/mL, ^1^/_8_ X = 1.95 μg/mL, ^1^/_4_ X = 0.482 μg/mL, ^1^/_2_× = 7.8 μg/mL. The paired analysis between the optimized formula and drug suspension was conducted within each concentration. Statistical significance was assessed by Student's *t*-test, and all *p* values are shown. ns means non-significant.

The biofilm inhibitory effect of the optimum drug-loaded cubogel formulation was compared to that of the drug suspension by adopting the crystal violet method, at sub-MIC concentrations (specifically concentrations equal to ^1^/_16_, ^1^/_8_, ^1^/_4_, and ^1^/_2_ X, where X is the calculated MIC). The optimum formulation had a significantly higher biofilm inhibitory effect than the drug suspension against the tested strain at only ^1^/_4_, and ^1^/_2_ MIC concentrations (Student's *t*-test, *p* value <0.05) (Fig. 5). This enhanced antibiofilm activity at sub-inhibitory concentrations suggests that the optimized formula not only disrupts fungal growth but also interferes with early biofilm formation stages, likely due to improved mucosal retention, sustained drug release, and increased surface interaction with fungal communities.

#### 3.3.7 Animal studies

##### 3.3.7.1 Ex vivo permeation study

**Fig. 6:**
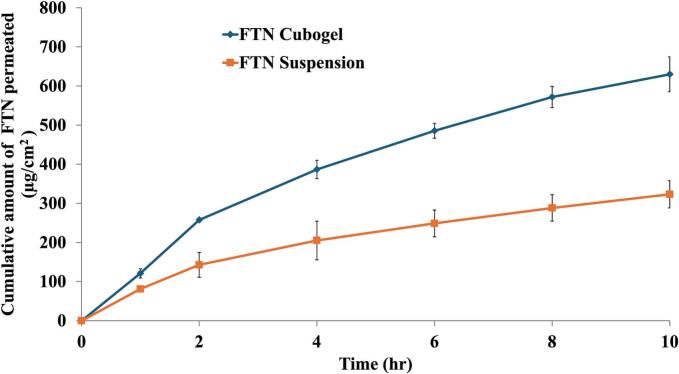
Permeation profile of fenticonazole from the optimum cubosomal formulation and its aqueous suspension, represented as cumulative amount of drug permeated against time.

Cumulative amount of FTN permeated through the tested vaginal membrane was plotted against time in Fig. 6. From the figure, it is evident that the amount of FTN permeated from the FTN-loaded cubogel is nearly double that permeated from the drug suspension after 10 h (629.95±44.54 $\mathrm{\mu g}/{\mathrm{cm}}^{2}$ vs 323.21±34.99 $\mathrm{\mu g}/{\mathrm{cm}}^{2}$, respectively). More specifically, FTN flux ($J_{\max}$) for the cubogel was significantly (*p* < 0.05) higher (63±4.45 $\mathrm{\mu g}/{\mathrm{cm}}^{2}/h$) than that of the FTN suspension (32.32±3.5 $\mathrm{\mu g}/{\mathrm{cm}}^{2}/h$). Overall, the cubogel possessed a permeation ER of 1.95 compared to that of the drug suspension. The enhancement in vaginal permeation can be attributed to number of reasons. For instance, the utilization of nanoparticle drug delivery system allowed for enhanced uptake through the membranes owing to the small particle sizes and larger surface areas. Cubosomes, in particular, arc characterized by mucoadhesive properties, due to the abundance of the hydrophilic hydroxyl groups within their backbones. This facilitates the interaction with several groups constituting the surface of the biological membranes which in turn extends contact time between the formulation and the membranes (Aboud et al., 2018; Sivadasan et al., 2023). Faisal et al. showed comparable findings when verapamil-loaded cubosomes yielded an ER of 2.26 compared to verapamil solution (Faisal et al., 2024). Additionally, GMO is a known permeation enhancer as well as moderate mucoadhesive (Lim et al., 2014; Tarsitano et al., 2022). Furthermore, T80 adds up to the permeation effect through enhancing drug solubility, increasing membrane porosity, as well as the fluidity of the nanovesicle through the biological membranes (Amirilargani et al., 2009; Rabiee et al., 2016; Saitoh et al., 2023). The incorporation of natural terpenes enhances drug permeation by interacting with biological membranes through multiple mechanisms. These compounds may fluidize membrane lipids and disrupt their structural integrity, thereby facilitating increased drug transport across the mucosal barrier. Additionally, they interfere with the lipid bilayers of the biological membranes through hydrogen bond formation, leading to disruption of the highly ordered structure of the stratum corneum. This results in increased lipid fluidity and improved diffusivity of the encapsulated drug, ultimately enhancing its permeation and therapeutic efficacy (Ahmed et al., 2025; Ahmed et al., 2024a). Taken together, these findings strongly support that the optimized cubogel significantly enhances vaginal permeation through a synergistic mechanism involving nanoscale delivery, mucoadhesion, membrane interaction, and functional excipients—thereby improving the therapeutic potential of FTN in local vaginal therapy.

##### 3.3.7.2 Confocal laser scanning microscopy (CLSM)

**Fig. 7:**
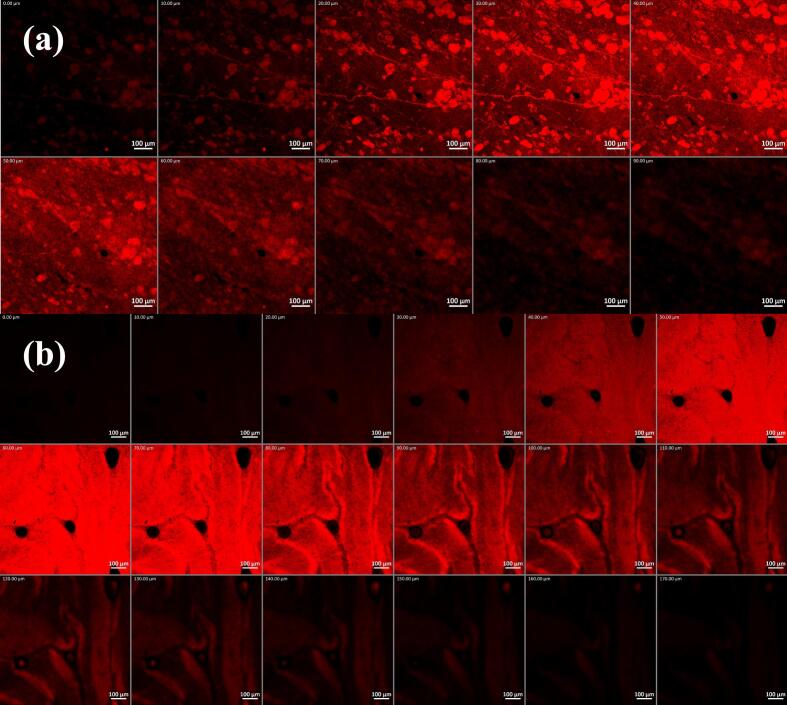
Confocal laser scanning microscopic images of (a) rhodamine B aqueous solution and (b) rhodamine B-loaded cubogel. The images suggest the deeper penetration of the cubogel.

CLSM was performed to investigate the penetrability of the formulation through vaginal mucosa. As can be seen in Fig. 7., RhB from the cubogel was able to reach deeper vaginal mucosal tissues (170 μm; Fig. 7b.) while that of the aqueous solution only managed to reach up to 90 μm deep (Fig. 7a.). The higher penetrability from the prepared cubogel is in close agreement with the findings from the ex vivo permeation evaluation. Overall, the nano nature of the embedded cubosomes, its mucoadhesive properties, as well as the permeation enhancing capabilities of both GMO and T80 on membrane porosity resulted in an increase in contact area, time, and permeation through vaginal mucosa. The result of ex vivo permeation is confirmed by the scans of CLSM, and both revealed the potential in vivo benefits of the cubogel. These CLSM findings further reinforce the effectiveness of the cubogel system in enhancing mucosal delivery by enabling deeper tissue penetration, which is critical for eradicating fungal pathogens residing within the deeper epithelial layers—thereby highlighting its promise as a superior alternative to conventional vaginal antifungal therapies.

##### 3.3.7.3 Histopathological assessment

**Fig. 8:**
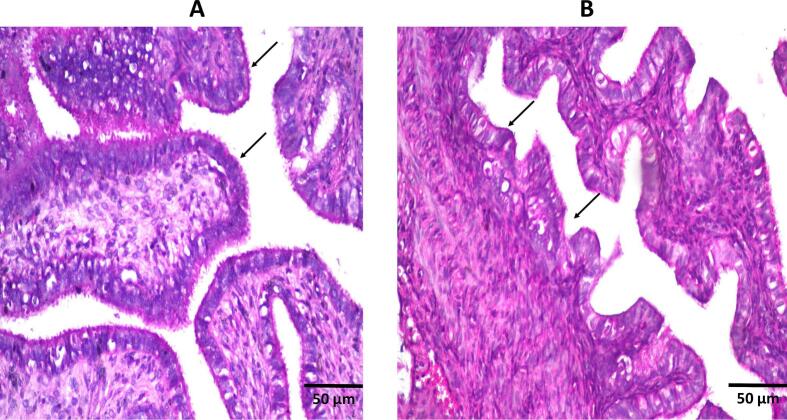
Histopathological images of (a) control group (left untreated) and (b) treatment group (drug-loaded cubogel), showing lack of alteration in normal tissue structure in both groups following staining with hematoxylin and eosin.

The results of histopathology (Fig. 8.) demonstrated the lack of differentiating items between the two groups (control and drug-loaded groups). Both groups show proper tissue structure of the vaginal epithelium and the underlying connective tissue. From the figure, normal vaginal mucosa structure was visible without any histopathological alteration. As a result of hematoxylin and eosin staining, the prepared FTN cubogel was deemed safe for vaginal application. These findings confirm the biocompatibility of the developed cubogel, indicating that the selected excipients and nanosystem components did not provoke any detectable local irritation, inflammation, or structural damage—supporting its suitability for repeated intravaginal administration.

## 4 Conclusion and future perspectives

The application of nanotechnology has tackled many obstacles related to conventional drug delivery approaches in a plethora of medical conditions. Vaginal candidiasis is a serious condition which presents a great deal of discomfort to a large proportion of women population with high chances of recurrence. Additionally, the current medication suffers from major limitation related to the low aqueous solubility, poor bioavailability, discomfort of vaginal discharges and/or systemic side effects. To circumvent such limitations, FTN cubosomes were prepared and the optimum cubosomal formulation was loaded into carbopol based gel to facilitate patient adherence. The optimum FTN cubosomal formulation possessed high %EE (85.32±2.34 %), small PS (169±0.85 nm), narrow particle distribution range (PDI = 0.29±0.02), and good surface stability indicated by adequate ZP (−24.40±1.27 mV). Around 86.77±3.79 % FTN was released from the cubosomal formulation over an 8-h release period ensuring the enhanced release profile of the prepared cubosomes. The FTN-loaded cubogel demonstrated a shear-thinning behavior, improving application and hence patient compliance. Biofilm inhibitory study revealed the enhanced effects of the cubogel over the standard drug suspension. Furthermore, both CLSM and ex vivo permeation studies confirmed the vastly improved cellular uptake and drug permeation through vaginal mucosal membranes of recently born, female albino rabbits. Histopathological assessment proved the safety of the prepared cubogel to vaginal mucosal membranes, as demonstrated by the lack of inflammatory response or structural alterations upon tissue treatment and staining with hematoxylin and eosin. To conclude, the prepared FTN cubogel represents a promising approach to treat vaginal candidiasis with no known compromises on short-term formulation stability or tissue safety. Future research should investigate the effects of the long-term storage as well as various storage conditions on the stability of the prepared cubogel. Additionally, the porosity and mechanical properties of the prepared gel can be evaluated. Moreover, the synergistic effects of fenchone as antifungal, antibacterial, as well as permeation enhancer should be thoroughly investigated.

## CRediT authorship contribution statement

**Sadek Ahmed:** Writing – review & editing, Resources, Investigation, Formal analysis, Conceptualization. **Osama Saher:** Writing – review & editing, Resources. **Heba Attia:** Writing – original draft, Resources, Investigation, Formal analysis, Conceptualization. **Abdurrahman M. Fahmy:** Writing – review & editing, Resources, Conceptualization. **Islam M. Adel:** Writing – original draft, Conceptualization.

## Ethical approval

Animal studies followed our institutional IACUC, the Research Ethics Committee at the Faculty of Pharmacy, Cairo University, Egypt (PI 3772). Furthermore, study was conducted in full accordance with the ARRIVE guidelines for reporting in vivo experiments. Additionally, all animal procedures complied with the principles outlined in the Guide for the Care and Use of Laboratory Animals, published by the U.S. National Institutes of Health (NIH Publication No. 85–23, revised 2011).

## Funding

The authors declare that no funds, grants, or other support were received during the preparation of this manuscript.

## Declaration of competing interest

The authors declare that they have no known competing financial interests or personal relationships that could have appeared to influence the work reported in this paper.

## Data Availability

The datasets generated during and/or analyzed during the current study are available from the corresponding author on reasonable request.
